# Protomer-Dependent Electronic Spectroscopy and Photochemistry of the Model Flavin Chromophore Alloxazine

**DOI:** 10.3390/molecules23082036

**Published:** 2018-08-14

**Authors:** Edward Matthews, Rosaria Cercola, Caroline E. H. Dessent

**Affiliations:** Department of Chemistry, University of York, Heslington, York YO10 5DD, UK; em645@york.ac.uk (E.M.); rc1274@york.ac.uk (R.C.)

**Keywords:** flavin, alloxazine, protomer, photodissociation spectroscopy, laser spectroscopy

## Abstract

Flavin chromophores play key roles in a wide range of photoactive proteins, but key questions exist in relation to their fundamental spectroscopic and photochemical properties. In this work, we report the first gas-phase spectroscopy study of protonated alloxazine (AL∙H^+^), a model flavin chromophore. Laser photodissociation is employed across a wide range (2.34–5.64 eV) to obtain the electronic spectrum and characterize the photofragmentation pathways. By comparison to TDDFT quantum chemical calculations, the spectrum is assigned to two AL∙H^+^ protomers; an N5 (dominant) and O4 (minor) form. The protomers have distinctly different spectral profiles in the region above 4.8 eV due to the presence of a strong electronic transition for the O4 protomer corresponding to an electron-density shift from the benzene to uracil moiety. AL∙H^+^ photoexcitation leads to fragmentation via loss of HCN and HNCO (along with small molecules such as CO_2_ and H_2_O), but the photofragmentation patterns differ dramatically from those observed upon collision excitation of the ground electronic state. This reveals that fragmentation is occurring during the excited state lifetime. Finally, our results show that the N5 protomer is associated primarily with HNCO loss while the O4 protomer is associated with HCN loss, indicating that the ring-opening dynamics are dependent on the location of protonation in the ground-state molecule.

## 1. Introduction

Flavins are redox-active chromophores that are widely found in animal and plant systems, where they play key roles in enzymes and photoreceptors [1,2,3]. Due to their extensive and complex biochemical roles, flavins have been the focus of many photophysical and photochemical studies, especially in aqueous solution [4,5,6]. These experimental studies have been supplemented over recent years by a growing number of computational studies [7,8,9,10,11,12]. However, gas-phase studies of the various electronic states of flavin chromophores are extremely sparse due to the experimental challenges of producing gaseous flavins [13,14], a situation which hampers a robust benchmarking of the computational work [7,8,9,10,11,12,15], as well as the fundamental understanding of the nature of the electronic transitions. There are hence a number of key underlying questions around the intrinsic flavin photophysics and photochemistry that remain unanswered [2]

**Figure 1:**
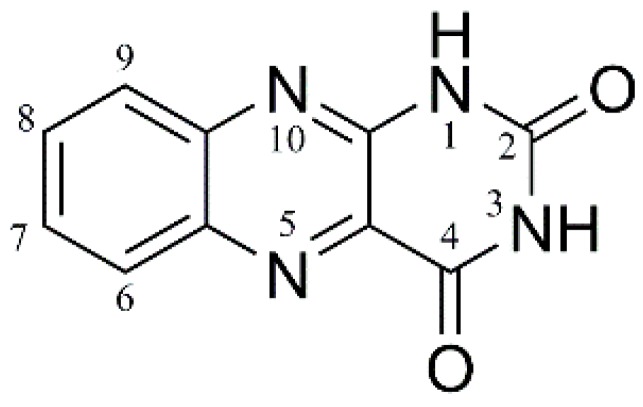
Schematic diagram of alloxazine, with atom labels.

In this work, we seek to begin to address this issue through studying the electronic laser spectroscopy and photochemistry of an isolated (i.e., gas-phase) model flavin chromophore, alloxazine, AL (Figure 1) across a wide visible and UV range. Alloxazine is very similar to lumichrome, which is the chromophore in flavins such as riboflavin and flavin mononucleotide, but lacks methyl groups at the 7 and 8 positions. This modest structural simplification means that AL is more amenable for high-level computational studies [9,12], leading us to select it for this study. We focus here on the protonated form of alloxazine, AL∙H^+^, which can be readily produced in the gas-phase via electrospray ionization.

In studying the protonated forms of multifunctional organic molecules such as alloxazine, it is clearly important to identify the location of the proton. A number of recent studies have focused on the identity of protonation isomers that are produced via electrospray, and it has been clearly established that a range of experimental factors (e.g., solvent and instrumentation) can affect the gaseous protomer distribution [16,17,18,19,20,21,22,23]. Importantly, there is no guarantee that the most stable solution-phase protomer will be the major gas-phase isomer following electrospray. We employ here the strategy we have successfully adopted recently for protonated nicotinamide and para-amino benzoic acid of identifying protonation isomers via a combination of low-resolution laser photodissociation spectroscopy coupled with computational chemistry [16,17].

The study of AL∙H^+^ presented here complements a very recent study by Shedrick et al., who have performed high-resolution photodissociation spectroscopy on the closely-related protonated lumichrome, LC∙H^+^ [24]. They obtained a vibronic spectrum across the region of the S_0_-S_1_ transition (485–502 nm), which they assigned to the N5-protonated isomer. Compared to this LC∙H^+^ study, the current work on AL∙H^+^ will provide insight into the electronic role of the LC methyl groups on the flavin chromophore. Our lower-resolution spectroscopic approach also allows us to obtain the electronic spectrum across a much wider spectral range, providing information on the higher electronic states as well as a broad insight into the UV photochemistry of the protonated flavin chromophore.

## 2. Results

### 2.1. Gas-Phase and Solution-Phase Absorption Spectroscopy of AL∙H^+^

**Figure 2:**
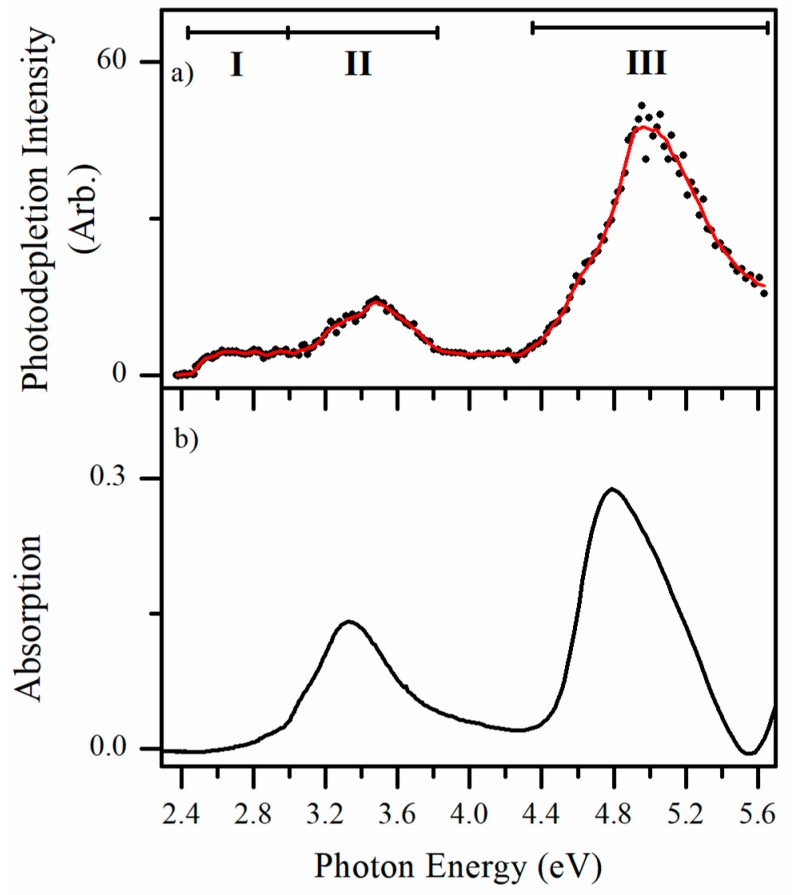
(**a**) Gas-phase photodepletion spectrum (absorption spectrum) of AL∙H^+^ across the range 2.34–5.64 eV (220–530 nm). The solid line is a five-point adjacent average of the data points. (**b**) Absorption spectrum (~1 × 10^−4^ mol dm^−3^ in MeOH/H_2_O) of alloxazine at pH = −0.9.

Electrospray ionization of alloxazine solutions readily produces AL∙H^+^ (*m*/*z* 215). The experiments presented here were conducted in a laser-interfaced mass spectrometer that is described in detail in Section 4. Figure 2a presents the gas-phase photodepletion spectrum of mass-selected AL∙H^+^ across the 2.38−5.64 eV range. In the limit where fluorescence is negligible, the photodepletion spectrum is equivalent to the gas-phase absorption spectrum, as discussed in detail previously [25,26,27,28]. AL∙H^+^ displays strong photodepletion in both the VIS and UV regions, with a photodepletion onset around 2.45 eV. The spectrum can be described as being split into two regions: a relatively weaker, VIS/UVA absorption region between 2.4–3.8 eV which is composed of two separate bands (**I**) and (**II**); and a stronger UVB/UVC region, which is composed of a broad band peaking around 5.0 eV (**III**). The rather flat profile of band **I** suggests considerable (unresolved) vibrational excitation for this band [24]. To our knowledge, this is the first gaseous absorption spectrum of a flavin chromophore that has been acquired by photodepletion. For comparison, Figure 2b displays the solution-phase absorption spectrum of AL; the spectrum agrees well with the previously published one [4].

It is evident that the solution spectrum of AL∙H^+^ broadly mirrors the gaseous absorption spectrum, which is important as it signifies that the gas-phase photodepletion spectrum is associated with single-photon dissociation of the ion. However, the band **I** feature of the gaseous ion spectrum appears substantially quenched (or possibly shifted) in the solution-phase spectrum, suggesting that this electronic transition is strongly affected by solvent interaction. In addition, the main absorption band (**III**) appears to be substantially red-shifted on moving from the gas-phase to solution (~0.3 eV). We return to identifying the protomeric species of AL∙H^+^ in Section 3.1.

### 2.2. Fragmentation of AL∙H^+^

#### 2.2.1. Higher-Energy Collisional Dissociation

**Figure 3:**
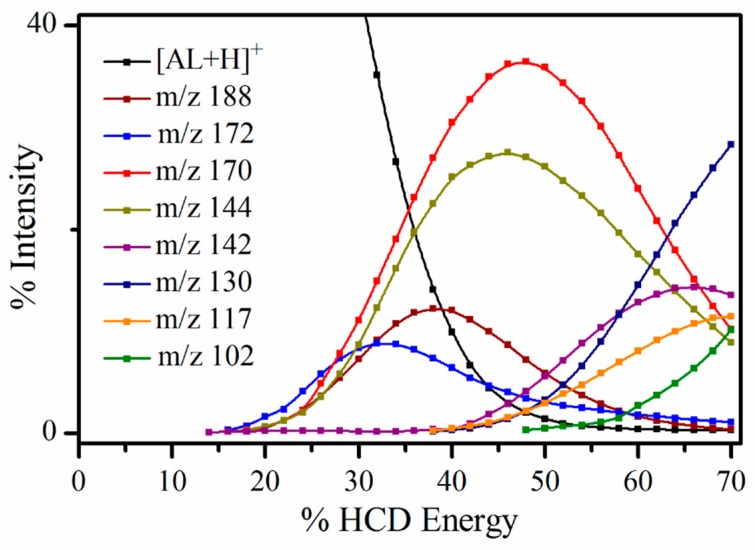
Parent ion dissociation curve AL∙H^+^ along with production curves for the eight most intense fragments upon HCD between 0 and 70% energy. The curved lines included with the data points are provided as a viewing guide, to emphasize the profile for an individual fragment.

Higher-energy collisional dissociation (HCD) was performed on AL∙H^+^ to fully characterize its ground electronic-state fragmentation behavior prior to conducting photoexcitation experiments. Figure 3 displays the HCD fragmentation curves which show that a number of molecular fragments are produced from AL∙H^+^, with most being strongly produced only within a limited energy range. As is generally the case, fragmentation into the lightest cationic species becomes dominant at the highest collision energies. At the very lowest collisional energies (<25%), the *m*/*z* 172 fragment is dominant, and is produced alongside *m*/*z* 170, 188 and 144. At intermediate collision energies (30–50% HCD), *m*/*z* 170 dominates, with *m*/*z* 144 also appearing strongly and with a similar fragmentation profile. Through this collisional range, the *m*/*z* 188 and 172 fragments are also produced, but with much lower intensities. At the highest energies, *m*/*z* 130 increases sharply, along with the other lighter fragment ions (*m*/*z* 142, 117 and 102). It is notable that all of the parent AL∙H^+^ is dissociated above 53%, so that all the fragments above this energy are secondary. Although we have not quantitatively calibrated the % HCD energies in Figure 3, previous work in our group has established that the 20–38% HCD energy range corresponds to internal energies of ~4–6 eV. [29]

Table 1 presents assignments of the HCD fragment ions, showing that the most intense fragments are produced by rupture of the pyrimidine ring and loss of small neutral molecules. Similar fragmentation patterns have been observed in electron impact ionization experiments on alloxazine-type molecules [30], where fragmentation was seen to begin with elimination of HNCO, followed by subsequent loss of CO, and then either HCN or CH_3_ units. 

#### 2.2.2. Photofragmentation Mass Spectroscopy

**Figure 4:**
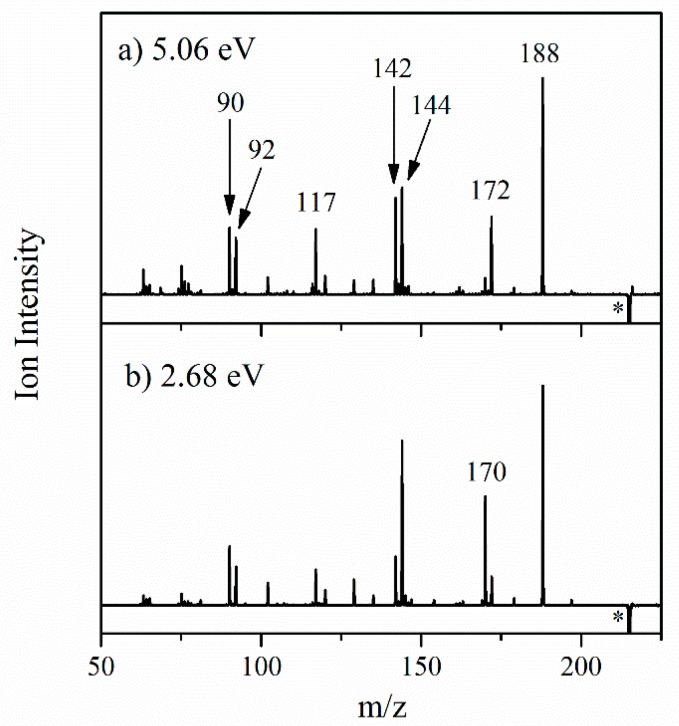
Photofragment difference (laser on–laser off) mass spectrum of AL∙H^+^, excited at (**a**) 5.06 eV (245 nm) and (**b**) 2.68 eV (463 nm). * indicates the depleted AL∙H^+^ ion signal.

We now turn to analyzing the cationic photofragments produced following photoexcitation to allow us to gain further insight into the nature of the excited states being accessed across the range of the photodepletion spectrum (Figure 2a). Figure 4 displays the difference (laser on–laser off) photofragment mass spectrum of AL∙H^+^, irradiated at the photodepletion maxima of 5.06 eV (band **III**) and 2.68 eV (band **I**), in the UVC and VIS regions, respectively.

In the UVC region (Figure 4a), the *m*/*z* 188, 172, 144 and 142 photofragments appear prominently. The *m*/*z* 90, 92 and 117 photofragments also have significant intensities, with a number of additional photofragments appearing with lower ion intensities. The pattern of fragmentation is similar upon VIS excitation (Figure 4b), with for example, fragments of *m*/*z* 188, 172, 170, 144, 142, and 117 appearing at both energies. However, some differences are evident, e.g., *m*/*z* 172 > *m*/*z* 170 at 5.06 eV, whereas *m*/*z* 170 > *m*/*z* 172 at 2.68 eV. A similar reversal in intensities at these two wavelengths is evident for the *m*/*z* 144 and 142 photofragments. Table 1 includes a list of the major photofragments observed at 5.06 and 2.68 eV, along with tentative assignments.

To properly interpret the fragmentation patterns observed going from 2.68 to 5.06 eV, it is important to compare the photoexcitation data to the HCD results. The photofragment production trends noted above for laser excitation at lower and higher excitation energies, does reflect the fragmentation patterns observed in HCD to some extent, especially in relation to *m*/*z* 142 which is only produced at very high collisional energies. However, it is clear that the photofragment intensities at both 2.68 and 5.06 eV do not match the fragment patterns produced across the entire HCD range. Furthermore, it is important that the dominant “anomalous” photofragment is the *m*/*z* 188 ion as it has the highest mass of all the photofragments produced, and cannot therefore be produced through any secondary dissociation process.

These observations indicate that photodissociation is non-statistical in both bands **I** and **III** [31], i.e., excited state decay does not occur by an ultrafast internal conversion followed by thermal decomposition on the ground state surface. This behavior is entirely different from the situation that we observed in our recent work on deprotonated ATP anions [29]. In that system, photoexcitation is largely localized on the adenine moiety, and is associated with ultrafast decay and statistical dissociation into fragments on the ground electronic state surface. The fragmentation patterns we obtained from HCD on the ATP anions, closely matched those of the photofragments.

#### 2.2.3. Photofragmentation Action Spectroscopy

**Figure 5:**
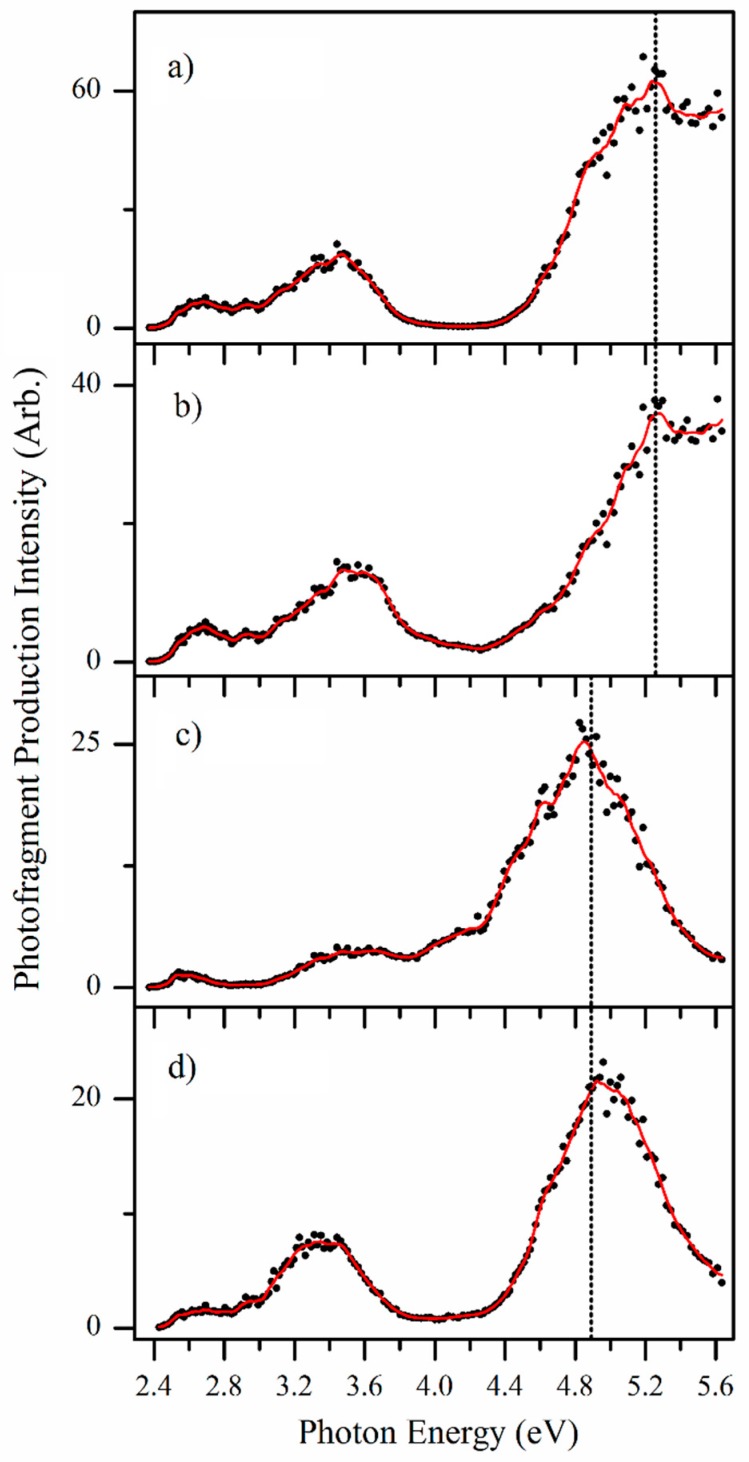
Photofragment action spectra of the fragments with (**a**) *m*/*z* 188, (**b**) *m*/*z* 144, (**c**) *m*/*z* 172 and (**d**) *m*/*z* 142 produced following photoexcitation of mass-selected AL∙H^+^ ions, across the range 2.34–5.64 eV. The solid line is a three-point adjacent average of the data points.

In the previous section, we focused on the photofragments produced at single-photon energies in the regions of band **I** and band **III**, and we now turn to presenting photofragment action spectra obtained across the entire spectral range. These spectra, shown in Figure 5, provide a complete picture of the wavelength-dependent production of the prominent *m*/*z* 188, 172, 144 and 142 photofragments. 

Inspection of the action spectra presented in Figure 5 reveals that the spectral profiles of these fragments appear to fall into two categories: The heaviest fragment *m*/*z* 188 along with the *m*/*z* 144 fragment spectra (Figure 5a,b) peak at 5.25 eV, and show strong production intensity at the high-energy spectral edge, whereas the *m*/*z* 172 and 142 fragment spectra (Figure 5c,d) peak at 4.85 eV, with decreasing intensity towards high energies.

In previous studies, we found that distinctive photofragment action spectra for different groups of photofragments can signify the presence of isomeric species in the electrosprayed ensemble of ions [16,17]. Following a similar interpretation, the *m*/*z* 188 and 144 pair of fragments would then be assigned as the primary fragments associated with photoinduced decay of one protomer, which fragments into an initially formed *m*/*z* 188 fragment that subsequently loses CO_2_. This fragmentation sequence corresponds to the second possible assignment of the *m*/*z* 144 fragment suggested in Table 1. A second protomeric isomer would then fragment into the *m*/*z* 172 and 142 pair of photofragments, similarly related by loss of H_2_CO from the initially formed *m*/*z* 172 photofragment. Note that dissociation of *m*/*z* 188 into *m*/*z* 144 at higher internal energies, and similarly, *m*/*z* 172 into *m*/*z* 142, is entirely consistent with the HCD data.

The photofragment action spectra shown in Figure 5c,d closely resemble the overall gaseous absorption spectrum (Figure 2a), with all three spectra peaking at the band **III** maximum at ~4.9 eV. The gaseous absorption spectrum is quite different from the Figure 5a,b photofragment action spectra, particularly across the higher-energy region. These points indicate that the protomer which fragments into the *m*/*z* 172 and 142 pair of photofragments is the dominant protomer in the electrosprayed ensemble of ions.

One question that can arise in photodissociation spectroscopy is whether the observed photofragments are inter-related through photofragmentation of primary photofragments following absorption of a second photon. We investigated this possibility by isolating the photofragments in the ion-trap, and then subjecting them to laser interaction. A similar set of measurements were conducted on the photofragments using low-energy CID activation to probe what products arise when these primary photofragments have excess thermal energy [25,32]. These results are presented in Section S1 of the Supplementary Materials, and conclusively show that none of the primary photofragments observed here (i.e., the ones with action spectra displayed in Figure 5) show a propensity to photodissociate (or thermally dissociate) into a lower mass primary fragment. Thus, we conclude that the photofragment action spectra map the electronic spectra of two distinctive protomers.

## 3. Discussion

### 3.1. Assignment of the Protomers of AL∙H^+^

Quantum chemical calculations were performed on a number of AL∙H^+^ protonation isomers (Table 2). The N5 protomer is identified as the lowest-energy isomer in both the gas-phase and solution-phase, but in water the N10 protomer is predicted to be only slightly higher in energy than the N5 protomer. From our calculations, we therefore predict that the solution-phase absorption spectrum (Figure 2b) will contain contributions from both the N10 and N5 protomers. (The solution-phase calculations were conducted for water, rather than a water-methanol mixture as used to obtain the experimental solution-phase spectrum (Figure 3b), to simplify the calculations. We anticipate that this should have minimal effect on the computational results, particularly given that the relative energies in solution of the two lowest-energy protomers lie much lower in energy than the other isomers.) Figure S2 of the Supplementary Materials gives a simulated solution-phase absorption spectrum with contributions from both of these protomers, which is in good agreement with the experimental spectrum. We could find no discussion of the likely presence of two protomers in past studies of the solution-phase spectroscopy of either alloxazine or the related lumichrome molecule [4]. This is highly surprising given how much work has been performed on solution-phase flavin chromophores [33,34].

**Figure 6:**
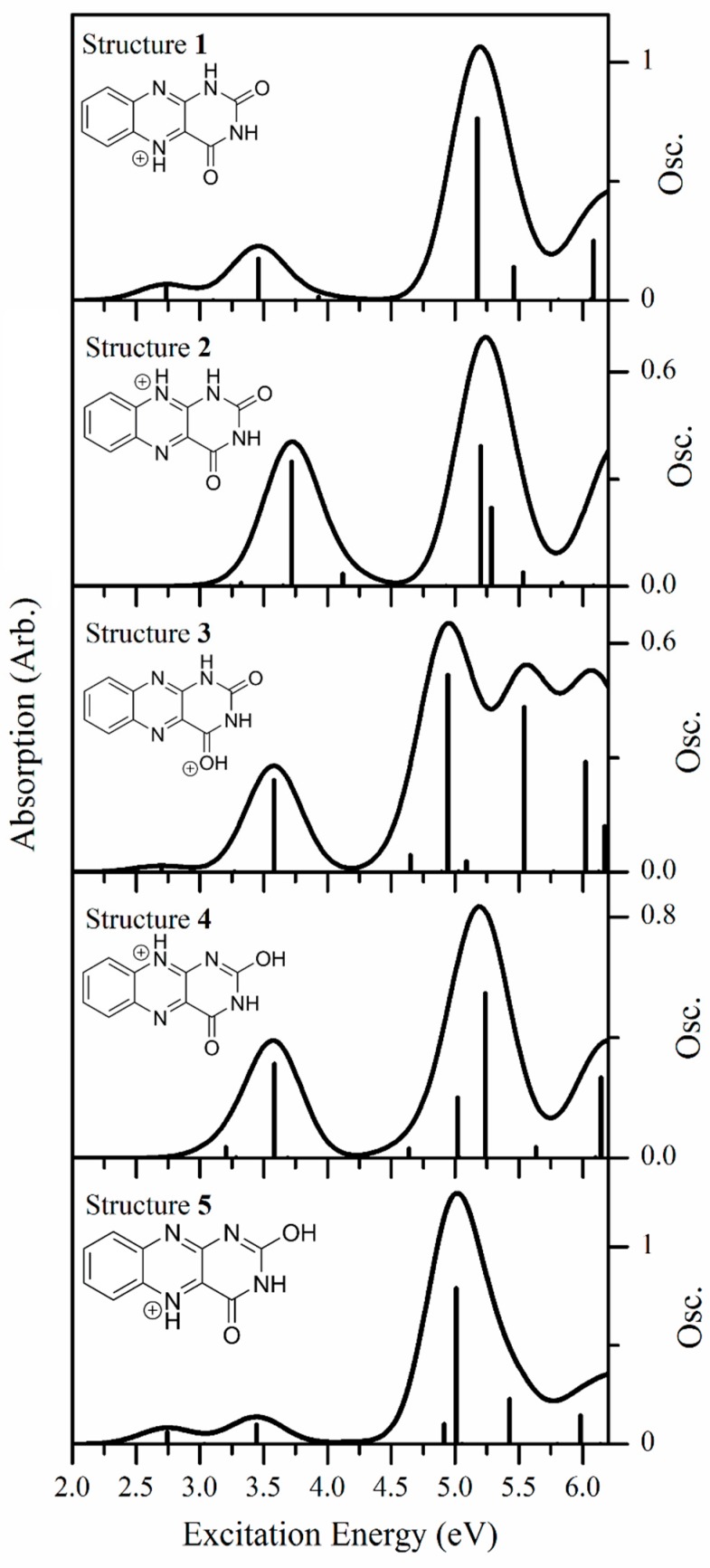
Calculated TDDFT excitation energies (with the PBE0 functional) of protomer **1**, protomer **2**, protomer **3**, protomer **4**, and protomer **5** of AL∙H^+^. The oscillator strengths (abbreviated Osc. on the y axis) of individual transitions are given by the vertical bars, while the full line spectrum is a convolution of the calculated spectrum with a Gaussian function (0.25 eV HWHM).

It is important to emphasize that these relative energy calculations cannot be used to straightforwardly predict which protomer(s) will be produced in the gas-phase. This has been well-established across a range of molecular systems and is attributed to the kinetic nature of the electrospray process [16,17,18,19,20,21,22,23]. Since the photofragment action spectra (Section 2.2.3) suggest the presence of two distinct electrosprayed protomers of AL∙H^+^, we performed TDDFT (Figure 6) calculations to identify them. Electronic spectra were calculated for all of the Table 2 protomers.

The spectra for all five protomers are broadly similar, in that they display low intensity absorption in the lower-energy VIS/UVA region, and then much stronger absorption bands in the higher-energy UVB/UVC region. Comparing the calculated spectra more closely, the spectra of isomers **1** and **5** appear very similar, with both displaying two bands in the low-energy region (with λ_max_ ~ 2.8 and 3.5 eV), followed by a stronger UVB/UVC region (with λ_max_ ~ 5.2 and 5.05 eV for protomers **1** and **5**, respectively). In addition, it is notable that there is a large separation between the VIS/UVA bands and the strong UVB/UVC bands for both protomers **1** and **5**, leading to an extended region between ~4.0 and 4.5 eV where the absorption is effectively zero. The similarity between the predicted electronic spectra of protomers **1** and **5** can be traced to the fact that both have H atoms at the N3 and N5 positions.

Similarly, the calculated spectra for protomers **2** and **4** closely resemble one another, due to both protomers having H atoms at the N3 and N10 positions. Each is predicted to display a UVA band with λ_max_ ~ 3.75 eV, and a UVB/UVC band at ~5.25 eV. The UVA and UVB/UVC bands are still well separated, but not as much so as for protomers **1** and **5**. Finally, the spectrum for protomer **3**, the only protomer with an H atom at the O4 position, is predicted to be highly distinctive over the higher-energy region, due to the presence of a pair of strong transitions with λ_max_ ~ 5.0 and 5.6 eV, leading to an almost continuously strong absorption across the high-energy region. Comparison of the gaseous photodepletion spectrum (Figure 2a) with the calculated spectra reveals that the shapes of the calculated protomer **1/**protomer **5** spectra (Figure 6) are both very similar to the experimental spectrum. In particular, the gaseous photodepletion spectrum displays two, significant intensity bands across the VIS/UVA region, which agrees well with the protomer **1/**protomer **5** calculated spectra. In addition, the calculated spectra of these protomers display the flat, “no-absorption” region around 4.0 eV seen in the experimental spectrum. The TDDFT calculations therefore provide good support for the presence of protomer **1** and/or **5** of AL∙H^+^ in the experimental ion ensemble. Given that protomer **5** is a substantially higher-energy isomer than protomer **1**, both in solution and the gas-phase, we believe that we can confidently assign the major protomer present in our experiment as protomer **1**. 

Next we turn to assigning the distinctive action spectra associated with the *m*/*z* 188 and 144 photofragments (Figure 5a,b). Comparing these spectra to the calculated TDDFT spectra suggests that protomer **3** is present, since both the experimental and calculated spectra display a distinctive region of strong and continuous absorption in the high-energy spectral region. As the overall photodepletion spectrum (Figure 2a) does not display this high-energy absorption feature, we can deduce that protomer **3** is a minor component of the gaseous ion ensemble. This is consistent with the calculated relative energies of protomer **1** and **3**. Therefore, analysis of our spectra leads us to conclude that two protomers are present, namely the N5 protomer (**1**) which dominates the electrosprayed sample, along with the O4 protomer (**3**).

Having assigned the photofragment action spectra, we note that protomer **1** fragments primarily via loss of HNCO, along with a fragment which we assign to loss of HNCO + H_2_CO, while protomer **3** fragments via loss of HCN and HCN + CO_2_. Intriguingly, these protomer **3** fragmentation channels were not identified in the electron impact ionization experiments, where the charge is believed to localize on an N atom (as in protomer **1** here) [30]. Therefore, the distinctive loss of HCN and HCN + CO_2_ we observe from protomer **3** here are consistent with production from the protonated carbonyl structure.

### 3.2. Nature of the Electronic Transitions of AL∙H^+^

**Figure 7:**
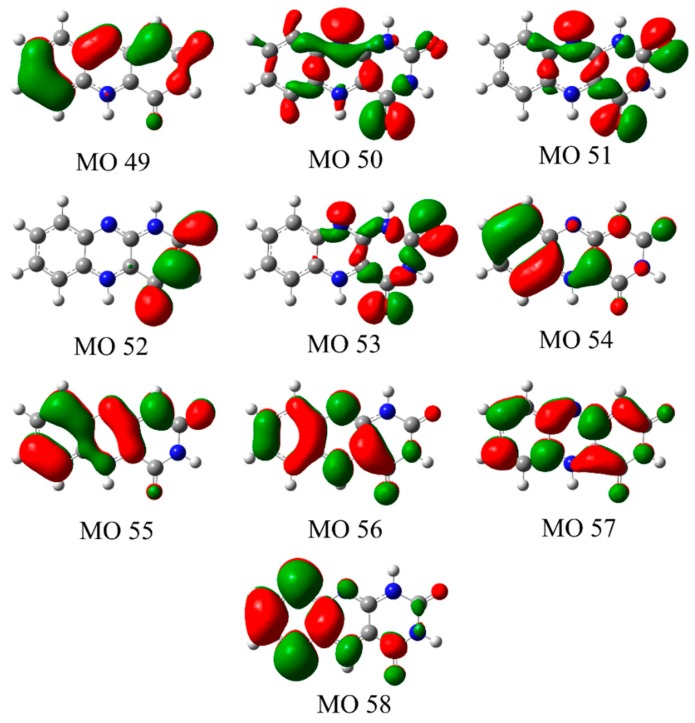
Molecular orbitals involved in the electronic transitions of protomer **1** (Table 3).

**Figure 8:**
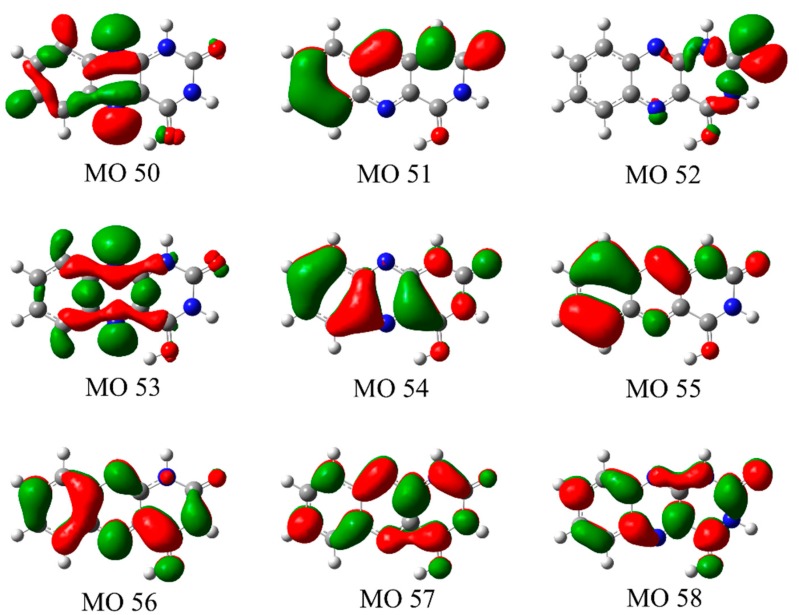
Molecular orbitals involved in the electronic transitions in protomer **3** of AL∙H^+^ (Table 4).

The energies, oscillator strengths, and the nature of the orbitals involved in the transitions for the calculated electronic transitions of the experimentally assigned protomers **1** and **3** are given in Table 3 and Table 4. For both protomers, the transitions with significant intensities correspond to π → π* transitions, with the transition intensities for the *n* → π* transitions being negligible in comparison. Our calculations are in line with previous analyses of the electronic spectra of alloxazines [4,5,8,9,10,11,12,15] which have stated that the *n* → π* transitions are hidden by the strong π → π* transitions for these species. Figure 7 and Figure 8 present the molecular orbitals involved in the dominant electronic transitions for protomers **1** and **3**. We note that the excess hydrogen in protomers **1** and **3** is bonded in-plane to the lone pair of a nitrogen or oxygen atom. As a result, the perpendicular π orbitals of protomers **1** and **3** possess similar shapes whereas the in-plane *n* orbitals are considerably different.

In the VIS/UVA region, the strong electronic transitions (bands **I** and **II** in Figure 2a) are predicted as π → π* transitions to the LUMO (orbital 56) in both of the protomers **1** and **3**. The experimental maximum of band **I** occurs at 2.68 eV, an excitation energy that is well reproduced by the calculations of protomers **1** and **3**, with the HOMO (orbital 55) to LUMO (orbital 56) transitions in protomers **1** and **3** being predicted to occur at 2.77 and 2.74 eV, respectively. For these transitions, excitation is predicted to correspond to a change in electron density within the uracil ring from the N1 and O2 to the N3 and O4 positions, along with an increase in electron density at the N5 position. It is notable that this is the protonation site for protomer **1**, providing an explanation for the significant (unresolved) vibrational excitation that appears to be present in band **I [24]**. An excitation-induced shift in electron density at this position would induce a geometry change along this coordinate. Band **II** of the gaseous absorption spectrum (3.5 eV) is reasonably well reproduced by the HOMO-1 (orbital 54) to LUMO (orbital 56) transition energy for both protomers **1** and **3**, with this transition being predicted to correspond to electron density being transferred to the N5 and N10 positions of the central ring. For protomer **1**, this change in electron density at the N5 position is again likely to lead to unresolved vibrational excitation along the coordinate of the protonation site.

The strongest electronic transition that occurs in the photodepletion spectrum (band **III**) occurs with a maximum of 5.06 eV (Figure 2a). Two electronic transitions from the HOMO and HOMO-1 to the LUMO+1 (orbital 57) are predicted in this region for both protomers **1** and **3**. The excited state orbital (orbital 57) for each protomer introduces an anti-bonding plane in the benzene-like ring when compared to the initial orbital, as well as having additional electron density on the uracil-like ring. The energy separation between these two predicted transitions is sufficiently low (<0.3 eV) that the transitions contribute to a single band at ~5 eV. For protomer **1**, the transitions contributing to the band at ~5 eV are the highest-energy strong transitions that are observed across the experimental spectral range (2.34–5.64 eV), resulting in a spectral profile that reproduces the experimental photodepletion band **III**. For protomer **3**, however, a strong transition (S_10_) between the orbital 55 (π) and orbital 58 (π*) is predicted to occur at 5.59 eV, characterized by the transfer of electron density from the benzene to the uracil ring. The excitation spectrum of protomer **3** is thus consistently elevated across the high-energy region of the spectrum.

### 3.3. Comparison of Protonated Lumichrome with Protonated Alloxazine

It is instructive to compare the work performed here to the recent study of Sheldrick et al. who used laser photodissociation spectroscopy to study the related protonated lumichrome molecule [24]. Their experiments were conducted across the narrower 19,700–20,800 cm^−1^ (2.442–2.579 eV) spectral range, on ions that were cryogenically cooled to 25 K. As in our experiment, they have identified an N5 protomer as the major isomeric species present. While they did not observe the O4 protomer, the spectral region studied in their higher-resolution work was outside of the region of the most intense O4 transitions seen here. It may also be the case that the minor O4 isomer was not formed in apparatus due to instrumental differences [21].

The higher-resolution measurements of Sheldrick et al. allowed them to identify the S_1_ origin transition as occurring at 19,962 cm^−1^ (2.475 eV), a value which is in the same region as the onset of band **I** in our photodepletion spectrum of AL∙H^+^ (~2.45 eV). The rather flat profile of band **I** in our AL∙H^+^ spectrum is also consistent with the extended vibronic progression observed by Sheldrick et al. for LC∙H^+^ associated with a large geometry change upon S_1_ excitation due to charge redistribution to the protonation site. These similarities indicate that the lack of methyl groups at the 7 and 8 positions of AL compared to LC appears to have limited electronic impact in the protonated system. 

## 4. Materials and Methods

UV photodissociation experiments were conducted in an AmaZon (Bruker, Billerica, MA, USA) ion-trap mass spectrometer that has been converted for laser experiments, as described in detail elsewhere [25,26]. AL∙H^+^ was generated by electrospraying a solution of AL in pure methanol (~1 × 10^−6^ mol dm^−3^). The AL was purchased from Santa Cruz Biotechnology (Dallas, TX, USA) and used without further purification.

AL∙H^+^ was mass selected and isolated in the ion-trap prior to laser irradiation. UV-VIS photons were produced by a 10 Hz, Surelite Nd:YAG (Amplitude Laser Group, San Jose, CA, USA) pumped Horizon, OPO (Amplitude Laser Group, San Jose, CA, USA) laser across the range 220–530 nm (2.34–5.64 eV). Scans were conducted using a 1 nm step size from 220–292, and with a 2 nm step size from 292–520 nm, with pulse energy ~1 mJ. Photofragmentation experiments were run with an ion accumulation time of 100 ms, and a fragmentation time of 100 ms, so that each mass selected ion packet interacted with just one laser pulse. This means that the probability of multiphoton excitation of an isolated parent ion (or a photofragment ion) is very low. (A laser power study at 462 nm verified that only a single photon was required to induce photofragmentation in this region.) The total absorbance of gaseous AL∙H^+^ is measured via photodepletion (PD) as a function of the scanned wavelength, with photofragment production (PF) also recorded at each wavelength [27]:

(1) $$\;\mathrm{Photodepletion}\;\mathrm{Intensity}\;=\;\frac{\mathrm{Ln}\left(\frac{{\mathrm{Int}}_{\mathrm{OFF}}}{{\mathrm{Int}}_{\mathrm{ON}}}\right)}{\lambda \;\times \;P}\;$$

(2) $$\;\mathrm{Photofragmentation}\;\mathrm{Production}\;=\;\frac{\left(\frac{{\mathrm{Int}}_{\mathrm{Frag}}}{{\mathrm{Int}}_{\mathrm{OFF}}}\right)}{\lambda \;\times \;P}\;$$

where Int_ON_ and Int_OFF_ are the peak intensities with laser on and off, Int_Frag_ is the fragment intensity with laser on, λ is the excitation wavelength (nm) and P is the laser pulse energy (mJ). Solution-phase absorption spectra were recorded in an 1800 UV spectrophotometer (Shimadzu, Kyoto, Kyoto Prefecture, Japan) using a 1 cm UV cuvette. 

Higher-energy collisional dissociation (HCD) was performed to investigate the ground-state fragmentation characteristics of AL∙H^+^. An Orbitrap Fusion Tribrid mass spectrometer (Thermo Fisher Scientific, Waltham, MA, USA) with an ESI source was employed for these experiments, run in positive ion mode. The HCD fragmentation technique as implemented on the Orbitrap mass spectrometer provides tandem mass spectrometry, similar to triple quadrupole fragmentation [35]. The instrument was operated with the following parameters: sweep gas flow rate, 0.1; sheath gas flow rate, 3.0; aux gas flow rate, 1.0; ion transfer tube temperature, 140 °C; vaporizer temperature, 36.3 °C; MS^1^ detector, Ion Trap; MS^1^ scan range, 50–600; MS^1^ maximum injection time, 100 ms; MS^2^ detector, Ion trap; MS^2^ maximum injection time, 100 ms. HCD collisional energy was varied between 0% and 70%.

All calculations were performed using Gaussian 09 [36], using density functional theory with the B3LYP, PBE0 and M062X functionals [37,38,39]. There are 20 possible protomers of AL∙H^+^, all of which were geometry optimized at the B3LYP/6-311+G* level initially. Only structures which had zero point energy corrected energies of less than 50 kJ mol^-1^ relative to the most stable structure were investigated in the subsequent calculations at the B3LYP/6-311++G(d,p) level. All protomers correspond to true minima as confirmed by frequency calculations, and were re-optimized using the polarized continuum model to approximate solvation effects. Time-dependent density functional theory (TDDFT) was used to calculate vertical excitation energies. Each TDDFT calculation used 30 states. A number of functionals were tested as part of this work (using the B3LYP/6-311++G(d,p) optimized structures), with results being reported only for the PBE0 functional in the main text, and for B3LYP and M062X in the Supplementary Materials [37,38,39]. PBE0 and B3LYP were found to perform best in TDDFT calculations of the related molecule, riboflavin, by Wu and Eriksson [15]. We note that the TDDFT calculations performed here are not expected to quantitatively reproduce the experimental spectra, but should provide a good qualitative guide for assigning the spectra. As part of these test calculations, we also explored the effect of including or removing the diffuse functions. Results were similar for the major excitations predicted in both cases, although when diffuse functions were included, all excitations to σ* final states appeared more prominently [40]. The oribital diagrams in Section 3.2 are for the set of calculations that do not include diffuse functions.

## 5. Conclusions

We have measured the gaseous electronic absorption spectrum and photofragment production spectra of protonated alloxazine, a model flavin chromophore, for the first time. Two protonation isomers are observed following electrospray ionization, corresponding to protonation at the N5 and O4 positions, and there are significant differences in the electronic spectra of these protomers. In particular, the O4 protomer displays a unique excitation in the high-energy spectral range (~5.6 eV) that our calculations predict is characterized by the transfer of electron density from the benzene to the uracil ring. It appears that protonation at the O4 position lowers the energy of the final-state orbital in this transition for the O4 protomer, so that it lies within the scanned spectral window only for this protomeric species.

The HCD measurements of AL∙H^+^ provide a detailed insight into the origins and energy-dependent formation of fragment ions. Ground-state AL∙H^+^ is observed to fragment via loss of small, stable neutral molecules such as HCN and HNCO. Our comparison of the HCD fragments with the photofragments produced following excitation of both bands **I** and **III** provides the first dynamical information on the isolated alloxazine system, revealing that photofragmentation is non-statistical and that excited state decay does not correspond to an ultrafast process. Intriguingly, this is true for both of the protomers observed in our experiment, as well as for photoexcitation across the various bands. Our results for AL∙H^+^ also mirror the results of Dugourd and co-workers who performed femtosecond pump-probe measurements on gaseous protonated flavin mononucleotide (FMN) [14]. They found that the UV photodissociation products differed dramatically from the collision-induced dissociation fragments. While the detailed fragmentation patterns are complex, and vary with probe pulse energy, an interesting result to emerge was that no changes to the photoproducts were observed upon varying the pump-probe timing from 0 to 100 ps. Like AL, this indicates that the FMN excited states are not collapsing back to the ground state on an ultrafast timescale

The work presented here should serve as a firm basis for performing time-resolved experiments to probe the dynamical events that follow photoexcitation. Our results clearly reveal that different dynamical processes occur for the different protomers, with protomer **1** being associated primarily with HNCO loss while protomer **3** is associated with HCN loss, indicating that different ring-opening processes occur for the different isomers. It is notable that HNCO loss is well-known to be associated with photoexcitation of single-ring uracil [41,42,43], and the AL∙H^+^ transition accessed close to 5 eV appears to be associated with almost localized excitation to the uracil moiety of AL. Although there have been a considerable number of experimental and theoretical studies to understand ring-opening dynamics over recent years [44], these studies have focused on single-ring systems. The tri-ring flavin group, present in the important biologically active flavins such as flavin adenine dinucleotide (FAD) and FMN, offers an important target system in which to extend these simpler studies. More generally, a full understanding of the electronic spectroscopy and photochemistry of the isolated chromophore is a crucial precursor to obtaining a better understanding of the intrinsic photophysics of FAD and FMN. Building on the work conducted here, current experiments are underway in our group to incrementally investigate these more complex systems.

## Figures and Tables

**Table 1:** Assignment of the fragmentation channels of AL∙H^+^ (*m*/*z* 215) observed upon HCD collisional excitation, and laser excitation at 2.68 and 5.06 eV. ^a,b^

Photofragment *m*/*z*	*m*/*z* Lost from AL∙H+	HCD	2.68 eV	5.06 eV	Photofragment Results from Loss of Neutral
188	27	✓	✓ (s)	✓ (vs)	HCN
172	43	✓	✓(w)	✓ (s)	HNCO ^a^
170	45	✓	✓ (s)	✓(w)	HCN + H_2_O
144	71	✓	✓ (s)	✓ (s)	(HNCO + CO) ^a^ Or, HCN + CO_2_
142	73	✓	✓(m)	✓ (s)	HNCO + H_2_CO Or, HCN + H_2_O + CO
130	85	✓	✓ (w)	✓ (w)	HNCO + NCO
117	98	✓	✓ (m)	✓ (s)	(HNCO + CO + HCN) ^a^
92	123	✓	✓ (s)	✓ (s)	HNCO + CO + (CN)_2_
90	125	✓	✓ (s)	✓ (s)	HNCO + CO + 2HCN

^a^ Fragmentation channels observed in electron impact ionization of alloxazine containing molecules [30]. ^b^ vs is very strong, s is strong, m is medium, and w is weak.

**Table 2:** Relative computed energies (gas-phase) of the optimized protomers of AL∙H^+^, calculated at the B3LYP/6-311++G(d,p) level. ^a,b^

Protomer	H1	H2	H3	Rel. E Gaseous (kJ mol^−1^)	Rel. E Water (kJ mol^−1^)
**1**	N1	N3	N5	0.0	0.0
**2**	N1	N3	N10	27.2	0.22
**3**	N1	N3	O4	19.5	36.3
**4**	O2	N3	N10	17.8	28.3
**5**	O2	N3	N5	37.1	50.4

^a^ The relative energies are zero-point energy corrected. ^b^ See Figure 1 for definitions of the atom labels.

**Table 3:** Singlet excitations and oscillator strengths (f) predicted by TDDFT (PBE0/6-311G(d,p)) calculations for protomer **1** of AL∙H^+^. Transitions < 5.8 eV included. MO transitions which contribute more than 20% are listed with the transition as well as an assignment of the initial and final orbitals.

State	Orbital Transitions	Δ*E* (eV)	f
S_1_	(0.92) 55(π) → 56(π*)	2.77	0.0542
S_2_	(0.84) 53(*n*) → 56(π*)	3.09	0.0004
S_3_	(0.85) 54(π) → 56(π*)	3.49	0.1650
S_4_	(0.61) 51(*n*) → 56(π*) + (0.25) 50(*n*) → 56(π*)	3.74	0.0003
S_5_	(0.99) 52(π) → 56(π*)	3.92	0.0129
S_6_	(0.72) 50(*n*) → 56(π*) + (0.23) 51(*n*) → 56(π*)	4.02	0.0000
S_7_	(0.81) 49(π) → 56(π*)	4.55	0.0017
S_8_	(0.81) 55(π) → 57(π*)	5.21	0.7409
S_9_	(0.87) 53(*n*) → 57(π*)	5.29	0.0000
S_10_	(0.66) 54(π) → 57(π*) + (0.23) 55(π) → 58(π*)	5.50	0.1594
S_11_	(0.61) 51(*n*) → 57(π*)	5.79	0.0000

**Table 4:** Singlet excitations and oscillator strengths (f) predicted by TDDFT (PBE0/6-311G(d,p)) calculations for protomer **3** of AL∙H^+^. Transitions <5.8 eV included. MO transitions which contribute more than 20% are listed with the transition as well as an assignment of the initial and final orbitals.

State	Orbital Transitions	Δ*E* (eV)	f
S_1_	(0.99) 55(π) → 56(π*)	2.74	0.0149
S_2_	(0.98) 53(*n*) → 56(π*)	3.28	0.0010
S_3_	(0.88) 54(π) → 56(π*)	3.62	0.2330
S_4_	(0.96) 52(*n*) → 56(π*)	4.51	0.0001
S_5_	(0.72) 51(π) → 56(π*) + (0.23) 55(π) → 57(π*)	4.69	0.0343
S_6_	(0.94) 50(*n*) → 56(π*)	4.92	0.0001
S_7_	(0.59) 55(π) → 57(π*) + (0.20) 51(π) → 56(π*)	5.02	0.5433
S_8_	(0.98) 53(*n*) → 57(π*)	5.04	0.0005
S_9_	(0.78) 54(π) → 57(π*)	5.14	0.0282
S_10_	(0.78) 55(π) → 58(π*)	5.61	0.3970

## Supplementary Materials

Supplementary materials are available on line.
