# Supplementary material for: Protomer-Dependent Electronic Spectroscopy and Photochemistry of the Model Flavin Chromophore Alloxazine
Source: Molecules. 2018 Aug 14;23(8):2036. doi: 10.3390/molecules23082036 (PMC6222404; doi:10.3390/molecules23082036)
Supplement: Supplementary file 1 [file molecules-23-02036-s001.pdf]

Supplementary Material

Protomer-Dependent Electronic Spectroscopy and Photochemistry of the Model Flavin Chromophore Alloxazine

Edward Matthews, Rosaria Cercola, and Caroline E. H. Dessent*

Department of Chemistry, University of York, Heslington, York, YO10 5DD, UK.

S1. Collision Induced Dissociation and Photofragmentation of the Major Photofragments formed from protonated alloxazine

S2. Simulation of the solution-phase absorption spectrum of protonated alloxazine

S3. Comparison of the PBE0, M06-2X and B3LYP TDDFT spectra of protonated alloxazine

S1. Collision Induced Dissociation and Photofragmentation Product Ions of the Major Photofragments formed from Protonated Alloxazine[a]

To explore the propensity of the major photofragments seen when protonated alloxazine is photoexcited (Figure 5) to fragment under thermal excitation and photoexcitation, the initial photofragments were isolated in the ion trap and then subjected to collision induced dissociation (CID) or photexcitation (248, 360 and 462 nm).

Table S1: CID and Photofragmentation Products from the Major Photofragments of protonated alloxazine.[a,b]

Photo-fragment m/z	CID Products	Photo-fragments Products (248 nm)	Photo-fragment Products (360 nm)	Photo-fragment Products (462 nm)
188	133, 145, 179, 117, 170	Very numerous, 133, 145	117, 135	-
170[c]	142, 188, 92	-	-	-
172	144	144, 117, 90	Very weak	-

[a] Fragments listed in decreasing order of intensity.

[b] Indicates that fragmentation was very weak.

[c] On isolation in the ion trap, m/z 170 forms a m/z 188 fragment corresponding to addition of water to m/z 170..

Section S2: Simulation of the solution-phase absorption spectrum of protonated alloxazine

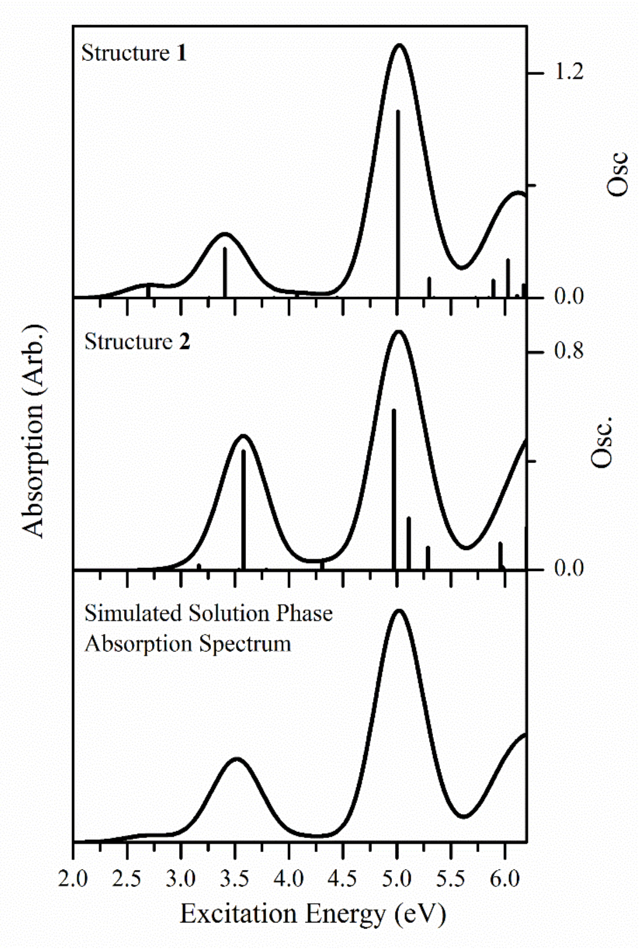

Figure S1: The top and middle spectra are the SCRF (Water) calculated TDDFT (PBE0/6-311++G(d,p)) excitation spectra of protomers **1** and **2** of AL·H^+, implicitly solvated in water. The bottom spectrum is the weighted-average excitation spectrum of protomers **1** and **2**, based on their Boltzmann distributions at 298K.

Section S3: Comparison of the PBE0, MO6-2X and B3LYP TDDFT spectra of protonated alloxazine

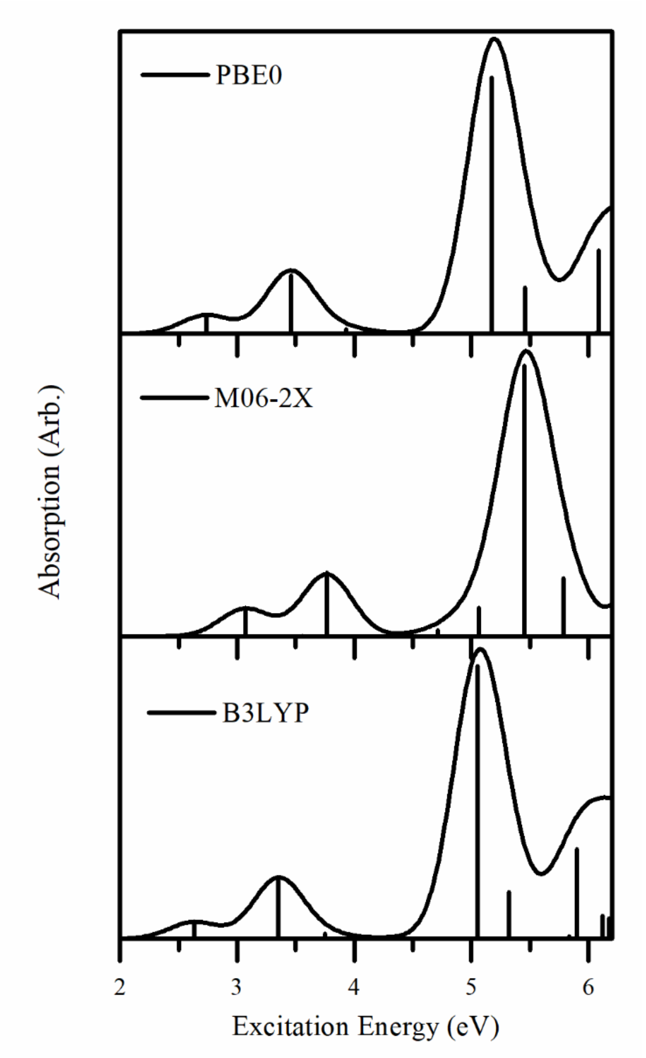

Figure S2: Calculated TDDFT excitation energies of protomer 1 of AL·H+ (see main text) with the PBE0 functional (top), the M06-2X functional (middle) and the B3LYP functional (bottom). The oscillator strengths of individual transitions are given by the vertical bars, while the full line spectrum is a convolution of the calculated spectrum with a Gaussian function (0.25 eV HWHM).
